# Supplementary material for: Heat adaptation of phage T7 under an extended genetic code
Source: Virus Evol. 2021 Dec 1;7(2):veab100. doi: 10.1093/ve/veab100 (PMC8923235; doi:10.1093/ve/veab100)
Supplement: veab100_Supp [file veab100_supp.zip › Sequencing_Analysis.zip!/Sequencing_Analysis/A2/evidence/JC_SIDE_1_54.html]

BRESEQ :: Evidence


| New junction evidence | | | | | | | | | | | |
| --- | --- | --- | --- | --- | --- | --- | --- | --- | --- | --- | --- |
|  | | seq id | position | reads (cov) | reads (cov) | score | skew | freq | annotation | gene | product |
| \* | ? | NC\_001604.1 | = 16476 | 362 (0.790) | 39 (0.090) | 31/590 | 11.1 | 9.2% | intergenic (–/–) | *–/–* | –/– |
|  | ? | NC\_001604.1 | 17143 = | 418 (0.930) |  |  |  |  | intergenic (–/–) | *–/–* | –/– |
| Rejected: Coverage evenness skew score above cutoff. | | | | | | | | | | | |
| Rejected: Frequency below/above cutoff threshold. | | | | | | | | | | | |

Only 100 of 367 total aligned reads displayed.  

`GCCTTGAATACCCTACTGCAATCTGCTGGTGCTCTCATCTGCAAACTGTGGATTATCAAGACCGAAGAGATGCTCGTAGAGAAAGGCTTGAAGCATGGCTGGGATGGGGACTTTGCGTACATGGCATGGGTACATGATGAAATCCAAGTAGGCTGCCGTACCGAAGAGATTGCTCAGGTGGTCATTGAGACCGCACAAGAAGCGATGCGCTGGGTTGGAGACCACTGGAACTTCCGGTGTCTTCTGGATACCGAAGGTAAGATGGGTCCTAATTGGGCGATTTGCCACTGATACAGGAGGCTACTCATGAACGAAAGACACTTAACAGGTGCTGCTTCTGAAATGCTAGTAGCCTACAAATTTACCAAAGCTGGGTACACTGTCTATTACCCTATGCTGACTCAGAGTAAAGAGGACTTGGTTGTATGTAAGGATGGTAAATTTAGTAAGGTTCAGGTTAAAACAGCCACAACGGTTCAAACCAACACAGGAGATGCCAAGCAGGTTAGGCTAGGTGGATGCGGTAGGTCCGAATATAAGGATGGAGACTTTGACATTCTTGCGGTTGTGGTTGACGAAGATGTGCTTATTTTCACATG  >  NC_001604.1/16177‑16775`  

                                                                                                                                                                                                                                                                                                           `|`                                                                                                                                                                                                                                                                                                             
`gCCTTGAATACCCTACTGCAATCTGCTGGTGCTCTCATCTGCAAACTGTGGATTATCAAGACCGAAGAGATGCTCGTAGAGAAAGGCTTGAAGCATGGCTGGGATGGGGACTTTGCGTACATGGCATGGGTACATGATGAAATCCAAGTAGGCTGCCGTACCGAAGAGATTGCTCAGGTGGTCATTGAGACCGCACAAGAAGCGATGCGCTGGGTTGGAGACCACTGGAACTTCCGGTGTCTTCTTGATACAGAAGGTAAGATGGGTCCTAATTGGGCGATTTCCCACTTATCAAGGTGGc                                                                                                                                                                                                                                                                                                            >  1:41342/1‑301 (MQ=255)`  
`gCCTTGAATACCCTACTGCAATCTGCTGGTGCTCTCATCTGCAAACTGTGGATTATCAAGACCGAAGAGATGCTCGTAGAGAAAGGCTTGAAGCATGGCTGGGATGGGGACTTTGCGTACATGGCATGGGTACATGATGAAATCCAAGTAGGCTGCCGTACCGAAGAGATTGCTCAGGTGGTCATTGAGACCGCACAAGAAGCGATGCGCTGGGTTGGAGACCACTGGAACTTCCGGTGTCTTCTGGATACCGAAGGTAAGATGGGTCCTAATTGGGCGATTTGCCACTGCTACAGGAGGc                                                                                                                                                                                                                                                                                                            >  1:686867/1‑301 (MQ=255)`  
  `cTTGAATACCCTACTGCAATCTGCTGGTGCTCTCATCTGCAAACTGTGGATTATCAAGACCGAAGAGATGCTCGTAGAGAAAGGCTTGAAGCATGGCTGGGATGGGGACTTTGCGTACATGGCATGGGTACATGATGAAATCCAAGTAGGCTGCCGTACCGAAGAGATTGCTCAGGTGGTCATTGAGACCGCACAAGAAGCGATGCGCTGGGTTGGAGACCACTGGAACTTCCGGTGTCTTCTGGATACCGAAGGTAAGATGGGTCCTAATTGGGCGATTTGCCACTGATACAGGAGGCTa                                                                                                                                                                                                                                                                                                          >  1:193129/1‑301 (MQ=255)`  
   `ttGAATACCCTACTGCAATCTGCTGGTGCTATCATCTGCAAACTGTGGATTATCAAGACCGAAGAGATGCTCGTAGAGAAAGGCTTGAAGCATGGCTGGGATGGGGACTTTGCGTACATGGCATGGGTACATAATGAAATCCAAGTAGGCTGCCGTACCGAAGAGATTGCTCAGGTGGTCATTGAGACCGCACAAGAAGCGATGCGCTGGGTTGGAGACCACTGGAACTTCCGGTGTCTTCTGGATACCGAAGGTAAGATGGGTCCTAATTGGGCGATTTGCCACTGATACAGGAGGCTAc                                                                                                                                                                                                                                                                                                         <  1:482690/301‑1 (MQ=255)`  
     `gAATACCCTACTGCAATCTGCTGGTGCTCTCATCTGCAAACTGTGGATTATCAAGACCGAAGAGATGCTCGTAGAGAAAGGCTTGAAGCATGGCTGGGATGGGGACTTTGCGTACATGGCATGGGTACATGATGAAATCCAAGTAGGCTGCCGTACCGAAGAGATTGCTCATGTGGTCATTGAGACCTCACAAGAAGCGATGCGCTGGGTTGGATACCACTGGAACTTCTGGTGTCTTCTGGATACCGAAGGTAAGATGGGTCCTAATTGGGCGATTTGCCACTGATACAGGAGGCTACTc                                                                                                                                                                                                                                                                                                       >  1:287351/1‑301 (MQ=255)`  
     `acagaCCCTACTGCAATCTGCTGGTGCTCTCATCTGCAAACTGTTGATTATCAAGACCGAATAGATGCTCGTAGAGAAAGGCTTGAAGCATGGCTGGGATGGGGACTTTGCGTACATGGCATGGGTACATGATGAAATCCAAGTAGGCTGCCGTACCTAAGAGATTGCTCAGGTGGTCATTGAGACCGCACAAGAAGCGATGCGCTGGGTTGTAGACCACTGGAACTTCCGGTGTCTTCTGGATACCGAATGTAAGATGGGTCCTAATTGGGCGATTTGCCACTGATACAGGAGGCTACTc                                                                                                                                                                                                                                                                                                       <  1:509666/297‑1 (MQ=255)`  
          `cccTACTGCAATCTGCTGGTGCTCTCATCTGCAAACTGTGGATTATCAAGACCGAAGAGATGCTCGTAGAGAAAGGCTTGAAGCATGGCTGGGATGGGGACTTTGCGTACATGGCATGGGTACATGATGAAATCCAAGTAGGCTGCCGTACCGAAGAGATTGCTCAGGTGGTCATTGAGACCGCACAAGAAGCGATGCGCTGGGTTGGAGACCACTGGAACTTCCGGTGTCTTCTGGATACCGAAGGTAAGATGGGTCCTAATTGGGCGATTTGCCACTTATACAGGAGGCTACTCATGaa                                                                                                                                                                                                                                                                                                  >  1:635944/1‑301 (MQ=255)`  
          `cccTACTGCAATCTGCTGGTGCTCTCATCTGCAAACTGTGGATTATCAAGACCGAAGAGATGCTCGTAGAGAAAGGCTTGAAGCATGGCTGGGATGGGGACTTTGCGTACATGGCATGGGTACATGATGAAATCCAAGTAGGCTGCCGTACCGAAGAGATTGCTCAGGTGGTCATTGAGACCGCACAAGAAGCGATGCGCTGGGTTGGAGACCACTGGAACTTCCGGTGTCTTCTGGATACCGAAGGTAAGATGGGTCCTAATTGGGCGATTTGCCACTGATACAGGAGGCTACTCATGaa                                                                                                                                                                                                                                                                                                  <  1:821923/301‑1 (MQ=255)`  
               `cTGCAATCTGCTGGTGCTCTCATCTGCAAACTGTGGATTATCAAGACCGAAGAGATGCTCGTAGAGAAAGGCTTGAAGCATGGCTGGGATGGGGACTTTGCGTACATGGCATTGGTACATGATGAAATCCAAGTAGGCTGCCGTACCGAAGAGATTGCTCAGGTGGTCATTGAGACCGCACAAGAAGCGATGCGCTGGGTTGGAGACCACTGGAACTTCCGGTGTCTTCTGGATACCGAAGGTAATATGGGTCCTAATTGGGCGATTTGCCACTGATACAGGAGGCTACTCATGAACGaaa                                                                                                                                                                                                                                                                                             >  1:700203/1‑301 (MQ=255)`  
                      `ctgctgGTGCTCTCATCTGCAAACTGTGGATTATCAAGACCGAAGAGATGCTCGTAGAGAAAGGCTTGAAGCATGGCTGGGATGGGGACTTTGCGTACATGGCATGGGTACATGATGAAATCCAAGTAGGCTGCCGTACCGAAGAGATTGCTCAGGTGGTCATTGAGACCGCACAAGAAGCGATGCGCTGGGTTGGAGACCACTGGAACTTCCGGTGTCTTCTGGATACCGAAGGTAAGATGGGTCCTAATTGGGCGATTTGCCACTGATACaggaggtgttctgactgtctcttatacac                                                                                                                                                                                                                                                                                      >  1:77027/1‑278 (MQ=255)`  
                         `ctgGTGCTCTCAACTGCAAACTGGGGAATATAAAGACCGAAGAGATGCTCGTAGAGAACGGCTTGAAGCATGGCTGGGATGGGGACTTGGCGTACATGGCATGGGTACATGATGAAATCCAAGTAGGCTGCCGTACCGAAGAGATTGCTCAGGTGGTCATTGAGACCGCACAAGAAGCGATGCGCTTGGTTGGAGACCACTGGAACTTCCGGTGTCTTCTGGATACCGAAGGTAAGATGGGTCCTAATTGGGCGATTTGCCACTGATACAGGAGGCTACTCATGAACGAAAGACACTTAAc                                                                                                                                                                                                                                                                                   <  1:126862/301‑1 (MQ=255)`  
                           `ggTGCTCTCATCTGCAAACTGTGGATTATCAAGACCGAAGAGATGCTCGTAGAGAAAGGCTTGAAGCATGGCTGGGATTGGGACTTTGCGTACATGGCATGGGTACATGATGAAATCCAAGTAGGCTGCCGTACCGAAGAGATTGCTCAGGTGGTCATTGAGACCGCACAAGAAGCGATGCGCTGGGTTGGAGACCACTGGCACTTCCGGTGTCTTCTGGATACCGCAGGTAAGATGGGTCCTAATTGGGCGATTTGCCACTGATACAGGAGGCTACTCATGAACGAAAGACACTTAACAg                                                                                                                                                                                                                                                                                 >  1:189430/1‑301 (MQ=255)`  
                              `gCTCTCATCTGCAAACTGTGGATTATCAAGACCGAAGAGATGCTCGTAGAGAAAGGCTTTAAGCATGGCTGGGATGGGGACTTTGCGTACATGGCATTGGTACATGATGAAATCCAAGTAGGCTGCCGTACCGAAGAGATTGCTCAGGTGGTCATTGAGACTGCACAAGAAGCGATGCGCTGGGTTGGAGACCACTGGAACTTCCGGTGTCTTCTGGATACCGAAGGTAAGATGGGTCCTAATTGGGCGATTTGCCACTGATACAGGAGGCTACTCATGAACGAAAGACACTTAACAGGtg                                                                                                                                                                                                                                                                              >  1:611107/1‑301 (MQ=255)`  
                               `ctctCATCTGCAAACTGTGGATTATCAAGACCGAAGAGATGCTCGTAGAGAAAGGCTTGAAGCATGGCTGGGATGGGGACTTTGCGTACATGGCATGGGTACATGATGAAATCCAAGTAGGCTGCCGTACCGAAGAGATTGCTCAGGTGGTCATTGAGACCGCACAAGAAGCAATGCGATGGGTTGGAGACCACTGGAACTTCCGGTGTCTTCTGGATACCGAAGGTAAGATGGGTCCTAATTGGGCGATTTGCCACTGATACAGGAGGCTACTCATGAACGAAAGACACTTAACAGGtgc                                                                                                                                                                                                                                                                             <  1:620679/301‑1 (MQ=255)`  
                               `ctctAATCTGCAAACTGTGGAGTATCAAGACCGAAGAGATGCTCGTAGAGAAAAGCTTGAAGCATGGCTGGGATGGGGACGTTGCGTACATGGCATGGGTACATGATGAAAGCAAAGTAGGCTGCCGTACCGAAGAGATTGCTCAGGTGGTCATTGAGACCGCACAAGAAGCGATGCGCTGGGTTGGAGACCACTGGAACTTCCGGTGTCTTCTGGATACCGAAGGTAAGATGGGTCCTAATTGGGCGATTTGCCACTGATACAGGAGGCTACTCATGAACGAAAGACACTTAACAGGtgc                                                                                                                                                                                                                                                                             <  1:347284/301‑1 (MQ=255)`  
                                   `cATCTGCAAACTGTGGATTATCAAGACCGAAGAGATGCTCGTAGAGAAAGGCTTGAAGCATGGCTGGGATGTGGACTTTGCGTACATGGCATGGGTACATGATGAAATCCAAGTAGGCTGCCGTACCGAAGAGATTGCTCAGGTGGTCATTGAGACCGCACAAGAAGCGATGCGCTGGGTTGGAGACCACTGGAACTTCCGGTGTCTTCTGGATACCGAAGGTAAGATGGGTCCTAATTGGGCGATTTGCCACTGATACAGGAGGCTACTCATGAACGAAAGACACTTAACAGGTgctgct                                                                                                                                                                                                                                                                         >  1:344204/1‑301 (MQ=255)`  
                                   `cATCTGCAAACTGTGGATTATCAAGACCGAAGAGATGCTCGTAGAGAAAGGCTTGAAGCATGGCTGGGATGGGGACTTTGCATACATGGCATGGGTACATGATGAAATCCAAGTAGGCTGACGTACCGAAGAGATTGCTCAGGTGGTCATTGAGACCGCACAAGAAGCGATGCGCTGGGTTGGAGACCACTGGAACTTCCGGTGTCTTCTGGATACCGAAGGTAAGATGGGTCCTAATTGGGCGATTTGCCACTGATACAGGAGGCTACTCATGAACGAAAGACACTTAACAGGTgctgct                                                                                                                                                                                                                                                                         >  1:648029/1‑301 (MQ=255)`  
                                         `cAAACTGTGGATTATCAAGACCGAAGAGATGCTCGTAGAGAAAGGCTTGAAGCATGGCTGGGATGGGGACTTTGCGTACATGGCATGGGTACATGATGAAATCCAAGTAGGCTGCCGTACCGAAGAGATTGCTCAGGTGGTCATTGAGACCTCACAAGAAGCGATGCGCTGGGTTGGAGACCACTGGAACTTCCGGTGTCTTCTGGATACCGAAGGTAAGATGGGTCCTAATTGGGCGATTTGCCACTGATACAGGAGGCTACTCATGAACGAAAGACACTTAACAGGTGCTGCTTCTGaa                                                                                                                                                                                                                                                                   <  1:272929/301‑1 (MQ=255)`  
                                             `cTGTGGATTATCAAGACCGAAGAGATGCTCGTAGAGAAAGGCTTGAAGCATGGCTTGGATGGGGACTTTGCGTACATGGCATGGGTACATGATGAAATCCAAGTAGGCTTCCGTACCGAAGAGATTGCTCAGGTGGTCATTGAGACCGCACAAGAAGCGATGCGCTGGGTTGGAGACCACTGGAACTTCCTGTGTCTTCTGGATACCGAAGGTAAGATGGGTCCTAATTTGGCGATTTGCCACTGATACAGGAGGCTACTCATGAACGAAAGACACTTAACAGGtgctgtctcttatacaa                                                                                                                                                                                                                                                               >  1:168309/1‑289 (MQ=255)`  
                                             `cTGTGGATTATCAAGACCGAAGAGATGCTCGTAGAGAAAGGCTTGAAGCATGGCTGGGATGGGGACTTTGCGTACATGGCATGGGTACATGATGAAATCCAAGTAGGCTGCCGTACCGAAGAGATTGCTCAGGTGGTCATTGAGACCGCACAAGAAGCGATGCGCTGGGTTGGAGACCACTGGAACTTCCGGTGTCTTCTGGATACCGAAGGTAAGATGGGTCCTAATTGGGCGATTTGCCACTGATACATGAGGCTACTCATGAACGAAAGACACTTAACAGGTGCTGCTTCTGAAATGc                                                                                                                                                                                                                                                               >  1:120348/1‑301 (MQ=255)`  
                                             `cTGTGGATTATCAAGACCGAAGAGATGCTCGTAAAGAAAGGCTTGAAGCATGGCTGGGATGGGGACTTTGCGTACATGGCCTGGGTACATGATGAAATCCAAGTAGGCTGCCGTACCGAAGAGATTGCTCAGGTGGTCATTGAGACCGCACAAGAAGCGATGCGCTGGGTTGGAGACCACTGGAACTTCCGGTGTCTTCTGGATACCGAAGGTAAGATGGGTCCTAATTGGGCGATTTGCCACTGATACCGGAGGCTACTCATGAACGAAAGACACATAACCGGtactgtctcttatacac                                                                                                                                                                                                                                                               >  1:175301/1‑289 (MQ=255)`  
                                               `gtgGATTATCAAGACCGAAGAGATGCTCGTATAGAAAGGCTTGAAGCATGGCTTGGATGGGGACTTTGCGTACATGGCATGGGTACATGATGAAATCAAAGTAGGATGCCGTACCGAAGAGATTGCTCAGGTGGTCATTGAGACCGCACAAGAAGCGATGCGCTGGGTTGGAGACCACTGGAACTTCCGGTGTCTACTGGATACCGAAGGTAAGATGGGTCCTAATTGGGCGATTTGCCACTGATACAGGAGGCTACTCATGAACGAAAGACACTTAACAGGtgcctgtctcttatacaca                                                                                                                                                                                                                                                             >  1:425523/1‑285 (MQ=255)`  
                                                    `ttATCAAGACCGAAGAGATGCTCGTAGAGAAAGGCTTGAAGCATGGCTGGGATGGGGACTTTGCGTACATGGCATGGGTACATGATGAAATCCAAGTAGGCTGCCGTACCGAAGAGATTGCTCAGGTGGTCATTGAGACCGCACAAGAAGCGATGCGCTGGGTTTGAGACCACTGGAACTTCCGGTGTCTTCTGGATACCGAAGGTAAGATGGGTCCTAATTGGGCGATTTGCCACTGATACAGGAGGCTACTCATGAACGAAAGCCACTTAACAggctgtctcttatacacatcttcgac                                                                                                                                                                                                                                                        >  1:746848/1‑277 (MQ=255)`  
                                                     `tATCAAGACCGAAGAGATGCTCGTAGAGAAAGGCTTGAAGCATGGCTGGGATGGGGACTTTGCGTACATGGCATGGGTACATGATGAAATCCAAGTAGGCTGCCGTACCGAAGAGATTGCTCAGGTGGTCATTGAGACCGCACAAGAAGCGATGCGCTGGGTTGGAGACCACTGGAACTTCCGGTGTCTTCTGGATACCGAAGGTAAGATGGGTCCTAATTGGGCGATTTGCAACTGATACAGGAGGCTACTCATGAACGAAAGACACTTAACAggctgtctcttatacacatctccgagc                                                                                                                                                                                                                                                       >  1:813567/1‑276 (MQ=255)`  
                                                     `gctcggagatgtgtataagagacaggagaAAGGCTTGAAGCATGGCTGGGATGGGGACTTTGCGTACATGGCATGGGTACATGATGAAATCCAAGTAGGCTGCCGTACCGAAGAGATTGCTCAGGTGGTCATTGAGACCGCACAAGAAGCGATGCGCTGGGTTGGAGACCACTGGAACTTCCGGTGTCTTCTGGATACCGAAGGTAAGATGGGTCCTAATTGGGCGATTTGCCACTGATACAGGAGGCTACTCATGAACGAAAGACACTTAACAGGTGCTGCTTCTGAAATGCTAGTAGcc                                                                                                                                                                                                                                                       <  1:10751/276‑1 (MQ=255)`  
                                                          `aGACTGAAGAGATGCTCGTAGAGAAAGGCTTGAAGCATGGCTGGGATGGGGACTTTGCGTACATGGCATGGGTACATGATGAAATCCAAGTAGGCTGCCGTACCGAAGAGATTGCTCAGGTGGTCATTGAGACCGCACAAGAAGCGATGCGCTGGGTTGGAGACCACTGGAACTTCCGGTGTCTTCTGGATACCGAAGGTAAGATGGGTCCTAATTGGGCGATTTGCCACTGATACAGGAGGCTACTCATGAACGAAAGACACTTAACAggctgtctcttatacacatctccgagcccacg                                                                                                                                                                                                                                                  >  1:537431/1‑271 (MQ=255)`  
                                                          `aGACCGAAGATATGCTCGTAGAGAAAGGCTTGAAGCATGGCTGGGATGGGGACTTTGCGTACATGGCATGGGTACATGATGAAATCCAAGTAGGCTGCCGTACCGAAGAGATTGCTCAGGTGGTCATTGAGACCGCACAAGAAGCGATGCGCTGGGTTGGAGACCACTGGAACTTCCGGTGTCTTCTGGATACCGAAGGTAAGATGGGTCCTAATTGGGCGATTTGCCACTGATACAGGAGGCTACTCATGAACGAAAGACACTTAACAGGtgcctgtctcttatacacatctccgagccc                                                                                                                                                                                                                                                  >  1:442735/1‑274 (MQ=255)`  
                                                           `gACCGAAGAGATGCTCGTATAGAAAGGCTTTAAGCATGGCTGGGATGGGGACTTTGCGTACATGGCATGGGTACATGATGAAATCCAAGTAGGCTGCCGTACCGAAGAGATTGCTCAGGTGGTCATTGAGACCGCACCAGAAGCGATGCGCTTGGTTGGAGACCACTGGAACTTCCGGTGTCTTCTGGATACCGAAGGTAAGATGGGTCCTAATTGGGCGATTTGCCACTGATACAGGAGGCTACTCATGAACGAAAGACACTTAACAGGTGCTGCTTCTGAAATGCTAGTAGCCTACaaa                                                                                                                                                                                                                                                 >  1:336904/1‑301 (MQ=255)`  
                                                           `gACCGAAGAGATGCTCGTAGAGAAAGGCTTGAAGCATGGCTGGGATGGGGACTTTGCGTACATGGCATGGGTACATGATGAAATCCAAGTAGGCTGCCGTACCGAAGAGATTGCTCAGGTGGTCATTGAGACAGCACAAGAAGCGATGCGCTGGGTTGGAGACCACTGGAACTTCCGGTGTCTTCTGGATACCGAAGGTAAGATGGGTCCTAATTGGGCGATTTGCCACTGATACAGGAGGCTACTCATGAACGAAAGACACTTAACAGGTGCTGCTTCTGAAATGCTAGTAGCCTACaaa                                                                                                                                                                                                                                                 >  1:697948/1‑301 (MQ=255)`  
                                                            `aCCGAAGAGATGCTCGTAGAGAAAGGCTTGAAGCATGGCTGGGATGGGGACTTTGCGTACATGGCATGGGTACATGATGAAATCCAAGTAGGCTGCCGTACCGAAGAGATTGCTCAGGTGGTCATTGAGACCGCACAAGAAGCGATGCGCTGGGTTGGAGACCACTGGAACTTCCGGTGTCTTCTGGATACCGAAGGTAAGATGGGTCCTAATTGGGCGATTTGCCACTGATACAGGAGGCTACTCATGAACGAAAGACACTTAACAGGTGCTGCTTCTGAAATGCTAGTAGCCTACAAAt                                                                                                                                                                                                                                                >  1:90846/1‑301 (MQ=255)`  
                                                                 `gagacagCTCGTAGAGAAAGGCTTGAAGCATGGCTGGGATGGGGACTTTGCGTACATGGCATGGGTACATGATGAAATCAAAGTAGGCTGCCGTACCGAAGAGATTGCTCAGGTAGTCATTGAGACCGCACAAGAAGCGATGCGCTGGGTTGGAGACCACTGGAACTTCCGGTGTCTTCTGGATACCGAAGGTAAGATGGGTCCTAATTGGGCGATTTGCCACTGATACAGGAGGCTACTCATGAACGAAAGACACTTAACAGGTGCTGCTTCTGAAATGCTAGTAGCCTACAAATTTAcc                                                                                                                                                                                                                                           <  1:305459/295‑1 (MQ=255)`  
                                                                    `gaTGCTCGTAGAGAAAGGCTTGAAGCATGGCTGGGATGGGGACTTTGCGTACATGGCATGGGTACATGATGAAATCCAAGTAGGCTGCCGTACCGAAGAGATTGCTCAGGTGGTCATTGAGACCGCACAAGAAGCGATGCGCTGGGTTGGAGACAACTGGAACTTCAGGTGTCTTCTGGATACCGAAGGTAAGATGGGTCCTAATTGGGCGATTTGACAATGATACAGGAGGCTACTCATGAACGAAAGACACTTAACAGGTGCTGCTTCTGAAATGATAGTAGCCAACAAATTTACCaaa                                                                                                                                                                                                                                        >  1:439240/1‑301 (MQ=255)`  
                                                                          `cGTATAGAAAGGCTTGAAGCATGGCTGGGATGGGGACTTTGCGTACATGGCATGGGTACATGATGAAATCCAAGTAGGCTGCCGTACCGAAGAGATTGCTCAGGTGGTCATTGAGACCGCACAAGAAGCGATGCGCTGGGTTGGAGACCACTGGAACTTCCGGTGTCTTCTGGATACCGAAGGTAAGATGGGTCCTAATTGGGCGATTTGCCACTGATACAGGAGGCTACTAATGAACTAAAGACACTTAACAGGTGCTGCTTCTGAAATGCTAGTAGCCTACAAATTTACCAAAGCTggg                                                                                                                                                                                                                                  >  1:224860/1‑301 (MQ=255)`  
                                                                           `gTAGAGAAAGGCTTGAAGCATGGCTGGGATGGGGACTTTGCGTACATGGCATGGGTACATGATGAAATCCAAGTAGGCTGCCGTACCGAAGAGATTGCTCAGGTGGTCATTGAGACCGCACAAGAAGCGATGCGCTGGGTTGGAGACCACTGGAACTTCCGGTGTCTTCTGGATACCGAAGGTAAGATGGGTCCTAATTGGGCGATTTGCCACTGATACAGGAGGCTACTCATGAACGAAAGACACTTAACAGGTGCTGCTTCTGAAATGCTAGTAGctgtctcttatacacatctccgag                                                                                                                                                                                                                                 >  1:678926/1‑278 (MQ=255)`  
                                                                           `aaggagaAAGGGTTGAAGCATGGCTGGGATGGGGACTTTGCGTACATGGCATGGGTACATGATGAAATCCAAGTAGGCTGCCGTACCGAAGAGATTGCTCAGGTGGTCATTGAGACCGCACAAGAAGCGATGCGCTGGGTTGGAGACCACTGGAACTTCCGGTGTCTTCTGGATACCGAAGGTAAGATGGGTCGTAATTGGGCGATTTGCCACTGATACAGGAGGCTACTCATGAACGAAAGACACTTAACAGGTGCTGCTTCTGAAATGCTAGTAGCCTACAAATTTACCAAAGCTGGGt                                                                                                                                                                                                                                 <  1:545539/298‑1 (MQ=255)`  
                                                                             `atagaAAGGCTTGAAGCATGGCTGGGATGGGGACTTTGCGTACATGGCATTGGTACATGATGAAATCCAAGTAGGCTGCCGTACCGAAGAGATTGCTCAGGTGGTCATTGAGACCGCACAAGAAGCGATGCGCTGGGTTGTAGACCACTGGAACTTCCGGTGTCTTCTGGATACCGAAGGTAAGATGGGTCCTAATTGGGCGATTTGCCACTGATACAGGAGGCTACTCATGAACGAAAGACACTTAACAGGTGCTGCTTCTGAAATGCTAGTAGCCTACAAATTTACCAAAGCTGGGTac                                                                                                                                                                                                                               <  1:430067/299‑1 (MQ=255)`  
                                                                              `gagaAAGGCTTGAAGCATGGCTGGGATGGGGACTTTGCGTACATGGCATGGGTACATGATGAAATCCAAGTAGGCTGCCGTACCGAAGAGATTGCTCAGGTGGTCATTGAGACCGCACAAGAAGCGATGCGCTGGGTTGGAGACCACTGGAACTTCCGGTGTCTTCTGGATACCGAAGGTAAGATGGGTCCTAATTGGGCGATTTGCCACTGATACAGGAGGCTACTCATGAACGAAAGACACTTAACAGGTGCTGCTTCTGAAATGCTAGTAGCCTACAAATTTACAAAAGCTGGGTaca                                                                                                                                                                                                                              >  1:110639/1‑301 (MQ=255)`  
                                                                                  `gatgtgtataagagacaggggATGGGGACTTTGCGTACATGGCATGGGTACATGATGGAATCCAAGTAGGCTGCCGTACCGAAGAGATTGCTCAGGTGGTCATTGAGACAGCACAAGAAGCGATGCGCTGGGTTGGAGACCACTGGCACTTCCGGTGTCTTCTGGATACCGAAGGTAAGATGGGTCCTAATTGGGCGATTTGCCACTGATACAGGAGGCTACTCATGAACGAAAGACACTTAACAGGTGCTGCTTCTGAAATGCTAGTAGCCTACAAATTTACCAAAGCTGCGTACACTGt                                                                                                                                                                                                                          <  1:294501/283‑1 (MQ=255)`  
                                                                                    `ggCTTGAAGCATGGCTGTGATGGGGACTTTGCGTAAATGGCATGGGTACATGATTAAATCCAAGTAGGCTGCCGTACCGAATAGATTGCACAGGTGGTCATTGAGACCGCACAAGAAGCGATGCGCTGGGTTGAATACCACTGGAACTTCCGGTGTCTTCTTGATACCGAAGGTAAGATGGGTCCTAATTGGGCGATTTGCCACTGATACAGGAGGCTACTCATGAACGAAAGACACTTAACAGGTGCTGCTTCTGAAATGCTAGTAGCCTACAAATTTACCAAAGCTGGGTACACTGTCt                                                                                                                                                                                                                        <  1:57814/301‑1 (MQ=255)`  
                                                                                    `ggCTTGAAGCATGGCTGGGATGGGGACTTTGCGTACATGGCATGGGTACATGATGAAATCCAAGTAGGCTGCCGTACCGAAGAGATTGCTCAGGTGGTCATTGAGACCGCACAAGAAGCGATGCGCTGGGTTGGAGACCACTGGAACTTCCGGTGTCTTCTGGATACCGAAGGTAAGATGGGTCCTAATTGGGCGATTTGCCACTGATACAGGAGGCTACTCATGAACGAAAGACACTTAACAGGTGCTGCTTCTGAAATGCTAGTAGCCTACAAATTTACCAAAGCTGGGTACACTGTCt                                                                                                                                                                                                                        >  1:752122/1‑301 (MQ=255)`  
                                                                                         `gggctcggagatgtgtataagagacaggTACATGGCATTGGTACATGATGAAATCCAAGTAGGCTTCCGTACCGAATAGATTGCTCAGGTGGTCATTGAGACCGCACAAGAATCGATGCGCTGGGTTGGAGACCACTGGAACTTCCGGTGTCTTCTGGATACCGAAGGTAAGATGGGTCCTAATTGGGCGATTTGCCACTGATACAGGAGGCTACTCATGAACGAAAGACACTTAACAGGTGCTGCTTCTGAAATGCTAGTAGCCTACAAATTTACCAAAGCTGGGTACACTGTCTATTAc                                                                                                                                                                                                                   <  1:391909/274‑1 (MQ=255)`  
                                                                                         `gAAGCATGGCTGGGATGGGGACTTTGTGTACATGGCATGGGTACATGATGAAATCCAAGTAGGCTGCCGTACCGAAGAGATTGCTCAGGTGGTCATTGAGACCGCACAAGAAGCGATGCGCTGGGTTGGAGACCACTGGAACTTCCGGTGTCTTCTGGATACCGAAGGTAAGATGGGTCCTAATTGGGCGATTTGCCACTGATACAGGAGGCTACTCATGAACGAAAGACACTTAACAGGTGCTGCTTCTGAAATGCTAGTAGCCTACAAATTTACCAAAGCTGGGTACACTGTCTATTAc                                                                                                                                                                                                                   <  1:311795/301‑1 (MQ=255)`  
                                                                                         `gAAGCATGGCTGGGATGGGGACTTTGCGTACATGGCATGGGTACATGATGAAATCCAAGTAGGCTGCCGTACCGAAGAGATTGCTCAGGTGGTCATTGAGACCGCACAAGAAGCGATGCGCTGTGTTGGAGACCACTGGAACTTCCGGTGTCTTCTGGATACCGAAGGTAAGATGGGTCCTAATTGGGCGATTTGCCACTGATACAGGAGGCTACTCATGAACGAAAGACACTTAACAGGTGCTGCTTCTGAAATGCTAGTAGCCTACAAATTTACCAAAGCTGGGTACACTGTCTATTAc                                                                                                                                                                                                                   <  1:531158/301‑1 (MQ=255)`  
                                                                                         `gAAGCATGGCTGGGATGGGGACTTTGAGTACATGGCATGGGTACATGATGAAATCCAAGTAGGCTGCCGTACCGAAGAGATTGCTCAGGTGGTCATTGAGACCGCACAAGAAGCGATGAGCTGGGTTGGAGACCACTGGAACTTACGGTGTCTTCTGGATACCGAAGGTAAGATGGGTCCTAATTGGGCGATTTGCCACTGATACAGGAGGCTACTCATGAACGAAAGACACTTAACAGGTACTGCTTCTGAAATGCTAGTAGCCTACAAATTTACCAAAGCTGGGTACACTGTCTATTAc                                                                                                                                                                                                                   <  1:376841/301‑1 (MQ=255)`  
                                                                                         `ctcggagatgtgtataagagacagtGCGTACATGGCATGGGTACATTATGAAATCCAAGTAGGCTGCCGTACCGAAGAGATTGCTCAGGTGGTCATTGAGACCGCACAAGAAGCGATGCGCTGGGTTGGAGACCACTGGAACTTCCGGTATCTTCTGGATACCGAAGGTAAGATGGGTCCTAATTGGGCGATTTGCCACTGATACAGGAGGCTACTCATGAACGAAAGACACTTAACAGGTGCTGCTTCTGAAATGCTAGTAGCCTACAAATTTACCAAAGCTGGGTACACTGTCTATTAc                                                                                                                                                                                                                   <  1:255683/277‑1 (MQ=255)`  
                                                                                          `gggagatgtgtataagggacagttGCGTACATGGCATGGGTACATGATGAAATCCAAGTAGGCTGCCGTACCGAAGAGATTGCTCAGGTGGTTATTGAGACCGCACAATAAGCGATGCGCTGGGTTGGAGACCACTGGAACTTCCGGTGTCTTCTGGATACCGAAGGTAAGATGGGTCCTAATTGGGCGATTTGCCACTGATACAGGAGGCTACTCATGAACGAAAGACACTTAACAGGTGCTGCTTCTGAAATGCTAGTAGCCTACAAATTTACCAAAGCTGGGTACACTGTCTATTAcc                                                                                                                                                                                                                  <  1:161708/279‑1 (MQ=255)`  
                                                                                           `tgggctcggagatgtgtataagagacagcatggcatggGTACATGATGAAATCCAAGTAGGCTGCCGTACCGAAGAGATTGCTCAGGTGGTCATTGAGACCGCACAAGAAGCGATGCGCTGGGTTGGAGACCACTGGCACTTCCTGTGTCTTCTGGATACCGAAGGTAAGATGGGTCCTAATTGGGCGATTTGCCACTGATACAGGAGGCTACTCATGAACGAAAGACACTTAACAGGTGCTGCTTCTGAAATGCTAGTAGCCTACAAATTTACCAAAGCTGGGTACACTGTCTATTAccc                                                                                                                                                                                                                 <  1:320307/273‑1 (MQ=255)`  
                                                                                           `agccttgCTGTGATGGGGACTTTGCGTACATTGCATGGGTACATGATGAAATACAAGTAGGCTGCAGTACCGAAGAGATTGCTCAGGTGGTCATTGAGACCGCAACAGAAGCGATGCGCTGGGTTGGAGACCACTGGAACTTCCGGTGTATTCTGGATACCGAAGGTAAGATGGGTCCTAATTGGGCGATTTGACACTGATACAGGAGGCTACTCATGAACGAAAGACACTTAACAGGTGCTGCCTCTGAAATGCTAGTAGCCTACAAATTTACCAAAGCTGGGTACACTGTCTATTAccc                                                                                                                                                                                                                 <  1:427983/295‑1 (MQ=255)`  
                                                                                           `aGCATGGCTGGGATGGGGACTTTGCGTACATGTCATGGGTACATGATGAAATCCAAGTAGGCTGCCGTACCGAATAGATTGCTCAGGTGGTCATTGAGACCGCACAAGAAGCGATGCGCTGGGTTGGAGACCACTGGAACTTCCGGTGTCTTCTTGATACCGAAGGTAAGATGGGTCCTAATTGGGCGATTTGCCACTGATACAGGAGGCTACTCATGAACGAAAGACACTTAACAGGTGCTGCTTCTTAAATGCTAGTAGCCTACAAATTTACCAAAGCTGGGTACACTGTCTATTAccc                                                                                                                                                                                                                 <  1:476382/301‑1 (MQ=255)`  
                                                                                                `tgtataagagacagcTTTGCGTACATGGCATGGGTACATGATGAAATCCAAGTAGGCTGCCGTACCGAAGAGATTGCTCAGGTGGTCATTGAGACCGCACAAGAAGCGATGCGCTGGGTTGGAGACCACTGGAACTTCCGGTGTCTTCTGGATACCGAAGGTAAGATGGGTCCTAATTGGGCGATTTGCCACTGATACAGGAGGCTACTCATGAACGAAAGACACTTAACAGGTGCTGCTTCTGAAATGCTAGTAGCCTACAAATTTACCAAAGCTGGGTACACTGTCTATTACCCTATGc                                                                                                                                                                                                            <  1:515182/287‑1 (MQ=255)`  
                                                                                                `ggCTGGGATGGGGACTTTGCGTACATGGCATGGGTACATGATGAAATCCAAGTAGGCTGCCGTACCGAAGAGATTGCTCAGGTGGTCATTGAGACCGCACAAGAAGCGATGCGCTGGGTTGGAGACCACTGGAACTTCCGGTGTCTTCTGGATACCGAAGGTAAGATGGGTCCTAATTGGGCGATTTGCCACTGATACAGGAGGCTACTCATGAACGAAAGACACTTAACAGGTGCTGCTTCTGAAATGCTAGTAGCCTACAAATTTACCAAAGCTGGGTACACTGTCTATTACCCTATGc                                                                                                                                                                                                            <  1:714210/301‑1 (MQ=255)`  
                                                                                                `ggCTGGGATGGGGACTTTGCGTACATGGCATGGGTACATGATGAAATCCAAGTAGGCTGCCGTACCGAAGAGATTGCTCAGGTGGTCATTGAGACCGCACAAGAAGCGATGCGCTGGGTTGGAGACCACTGGAACTTCCGGTGTCTTCTGGATACCGAAGGTAAGATGGGTCCTAATTGGGCGATTTGCCACTGATACAGGAGGCTACTCATGAACGAAAGACACTTAACAGGTGCTGCTTCTGAAATGCTAGTAGCCTACAAATTTACCAAAGCTGGGTACACTGTCTATTACCCTATGc                                                                                                                                                                                                            >  1:44514/1‑301 (MQ=255)`  
                                                                                                      `gATGGGGACTTTGCGTACATGGCATGGGTACATGATGAAATCCAAGTAGGCTGTCGTACCGAAGAGATTGCTCAGGTGGTCATTGAGACCGCACAAGAAGCGATGCGCTGGGTTGGAGACCACTGGAACTTCCGGTGTCTTCTGGATACCGAACGTAAGATGGGTCCTAATTGGGCGATTTGCCACTGATACAGGAGGCTACTCATGAACGAAAGACACTTAACAGGTGCTGCTTCTGAAATGCTAGTAGCCTACAAATTTACCAAAGCTGGGTACACTGTCTATTACCCTATGCTGACTc                                                                                                                                                                                                      >  1:811887/1‑301 (MQ=255)`  
                                                                                                      `gATGGGGACTTTGCGTACATGGCATGGGTACATGATGAAATCCAAGTAGGCTGCCGTACAGAAGAGATTGCTCAGGTGGTCATTGAGACCGCACAAGAAGCGATGCGCTGGGTTGGAGACCACTGGAACTTCCGGTGTCTTCTGGATACCGAAGGTAAGATGGGTCCTAATTGGGCGATTTGACACTGATACAGGAGGCTACTCATGAACGAAAGACACTTAACAGGTGCTGCTTCTGAACTGCTATTAGCCTACAAATTTACCAAAGCTGGCTACACTGTCTactgtctcttatacacat                                                                                                                                                                                                      >  1:522013/1‑284 (MQ=255)`  
                                                                                                          `gggACTTTGCGTACATGGCATGGGTACATGATGAAATCCAAGTAGGATGCCGTACCGAAGAGATTGCTCAGGTGGTCATTGAGACCACACAAGAAGCGATGCGCTGGGTTGGAGACCACTGGAACTTCCGGTGTCTTCTGGATACCGAAGGTAAGATGGGTCCTAATTGGGCGATTTGCCACTGATACAGGAGGCTACTCATGAACGAAAGACACTTAACAGGTGCTGCTTCTGAAATGCTAGTAGCCTACAAATTTACCAAAGCTGGGTACACTGTCTATTACCCTATGCTGACTCagag                                                                                                                                                                                                  <  1:137640/301‑1 (MQ=255)`  
                                                                                                              `cTTTGCGTACATGGCATGGGTACATGATGAAATCCAAGTAGGCTGCCGTACCGAAGAGATTGCTCAGGTGGTCATTGAGACCGCACAAGAAGCGATGCGCTGGGTTGGAGACCACTTGAACTTCCGGTGTCTTCTGGATACCGAAGGTAAGATGGGTCCTAATTGGGCGATTTGCCACTGATACAGGAGGCTACTCATGAACGAAAGACACTTAACAGGTGCTGCTTCTGAAATGCTAGTAGCCTACAAATTTACCAAAGCTGGGTACACTGTCTATTACCCTATGCTGACTCAGAGTaaa                                                                                                                                                                                              >  1:602831/1‑301 (MQ=255)`  
                                                                                                                   `gagatgtgtataagagaCAGGATGAAATCCAAGTAGGCTGCCGTACCGAAGAGATTGCTCAGGTGGTCATTGAGACCGCACAAGAAGCGATGCGCTGGGTTGGAGACCACTGGAACTTCCGGTATCTTCTGGATACCGAAGGTAAGATGGGTCCTAATTGGGCGATTTGCCACTGATACAGGAGGCTACTCATGAACGAAAGACACTTAACAGGTGCTGCTTCTGAAATGCTAGTAGCCTACAAATTTACCAAAGCTGGGTACACTGTCTATTACCCTATGCTGACTCAGAGTAAAGAGGa                                                                                                                                                                                         <  1:102854/285‑1 (MQ=255)`  
                                                                                                                       `catggcatggGTACATGATGAAATCCAAGTAGGCTGCCGTACCGAAGAGATTGCTCAGGTGGTCATTGAGACCGCACAAGAAGCGATGCGCTGGGTTGGAGACCACTGGAACTTCCGGTGTCTTCTGGATACCGAAGGTAAGATGTGTCCTAATTGGGCGATTTGCCACTGATACAGGAGGCTACTCATGAACGAAAGACACTTAACAGGTGCTGCTTCTGAAATGCTAGTAGCCTACAAATTTACCAAAGCTGGGTACACTGTCTATTACCCTATGcctgtctcttatacacatctccga                                                                                                                                                                                     >  1:552460/1‑278 (MQ=255)`  
                                                                                                                         `tggcatggGTACATGATGAAATCCAAGTAGGCTGCCGTACCGAAGAGATTGCTCAGGTGGTCATTGAGACCGCACAAGAAGCGATGCGCTGGGTTGGAGACCACTGGAACTTCCGTTGTCTTCTGGATACCGAAGGTAAGATGGGTCCTAATTGGGCGATTTGCCACTGATACAGGAGGCTACTCATGAATGAAAGACACTTAACAGGTGCTGCTTCTGAAATGCTAGTAGCCTACAAATTTACCAAAGCTGGGTACACTGTCTATTAcccctgtctcttatacacatctccgagcccacg                                                                                                                                                                                   >  1:672534/1‑271 (MQ=255)`  
                                                                                                                         `gggcatggGTACATGATGAAATCCAAGTAGGCTGCAGTACCGAAGAGATTGCTCAGGTGGTCATTGAGACCGCACAAGAAGCGATGCGCTGGGTTGGAGACCACTGGAACTTCCGGTGTCTTCTGGATACCGAAGGTAAGATGGGTCCTAATTGGGCGATTTGCCACTGATACAGGAGGCTACTCATGAACGAAAGACACTTAACAGGTGCTGCTTCTGAAATGCTAGTAGACTACAAATTTACCAAAGCTGGGTACACTGTCTATTACCCTATGCTGACTCAGAGTAAAGAGGACTTGGt                                                                                                                                                                                   <  1:742161/300‑1 (MQ=255)`  
                                                                                                                           `gcatggGTACATGATGAAATCCAAGTAGGCTGCCGTACCAAAGAGATTGCTCAGGTGGTCATTGAGACCGCACAAGAAGCGATGCGCTGGGTTGGAGACCACTGGAACTTCCGGTGTCTTCTGGATACCGAAGGTAAGATGGGTCCTAATTGGGCGATTTGCCACTGATACAGGAGGCTACTCATGAACGAAAGACACTTAACAGGTGCTGCTTCTGAAATGCTAGTAGCCTACAAATTTACCAAAGCTGGGTACACTGTCTATTACCCTATGCTGACTCAGAGTAAAGAGGACTTGGttc                                                                                                                                                                                 >  1:314821/1‑300 (MQ=255)`  
                                                                                                                              `tggGTACATGATGAAATCCAAGTAGGCTGCCATACCGAAGAGATTGCTCAGGTGGTCATTGAGACCGAACAAGAAGCGATGCGCTGGGTTGGAGACCACTGGCACTTCCGGTGTCTTCTGGATACCGAAGGTAAGATGGGTCCTAATTGGGCGATTTGCCACTGATACAGGAGGCTACTCATGAACGAAAGACACTTAACAGGTGCTGCTTCTGAAATGCTAGTAGCCTACAAATTTACCAAAGCTGGGTACACTGTCTATTACCCTATGCTGACTCAGAGTAAAGAGGACTTGGTtgtat                                                                                                                                                                              <  1:729968/301‑1 (MQ=255)`  
                                                                                                                                   `ccATGATGAAAGCCAAGTAGGGCGCCGTACCGAAGAGATTGCTCAGGTGGTCATTGAGACCGCACAAGAAGCGATGCGCTGGGTTGGAGACCACTGGAACTTCCGGTGTCTTCTGGATACCGAAGGTCAGATGGGTCCTAATTGGGCGATTTGCCACTGATACAGGAGGCTACTCATGAACGAAAGACACTTAACAGGTGCTGCTTCTGAAATGCTAGTAGCCTACAAATTTACCAAAGCTGGGTACACTGTCTATTACCCTATGCTGACTCAGAGTAAAGAGGACTTGGTTGTATGTAAg                                                                                                                                                                         <  1:630853/300‑1 (MQ=255)`  
                                                                                                                                    `cATGATGAAATACAAGTAGGCTGCCGTACCGAAGAGAGTGCTCAGGTGGTCATTGAGACCGCACAAGAAGCGATGCGCTGGGTTGGAGACCACTGGAACTTCCGGTGTCTTCTGGATACCGAAGGTAAGATGGGTCCTAATTGGGCGATTTGCCACTGATACAGGAGGCTACTCATGAACGAAAGACACTTAACAGGTGCTGCTTCTGAAATGCTAGTAGCCTACAAATTTACCAAAGCTGGGTACACTGTCTATTACCCTATGCTGACTCAGAGTAAAGAGGACTTGGTTGTATGTAAgg                                                                                                                                                                        <  1:362691/301‑1 (MQ=255)`  
                                                                                                                                             `aTCCAAGTAGGCTGCCGTACCGAAGAGATTGCTCAGGTGTTCATTGAGACCGCACAAGAAGCGATGCGCTGGGTTGGAGACCACTGGAACTTCCGGTGTCTTCTGGATACCGAAGGTAATATGGGTCCTAATTGGGCGATTTGCCACTGATACAGGAGGCTACTCATGAAAGAAAGACACTTAACAGGTGCTGCTTCTGAAATGCTAGTAGCCTACAAATTTACCAAAGCTGGGTACACTGTCTATTACCCTATGCTGACTCAGAGTAAAGAGGACTTGGTTGTATGTAAGGATGGTAAAt                                                                                                                                                               >  1:493643/1‑301 (MQ=255)`  
                                                                                                                                                             `gagtctcgtgggctcggagatgtgtataagagaCAGCACAAGAAGCGATGCGCTGGGTTGGAGACCACTGGAACTTCCGGTGTCTTCTGGATACCGAAGGTAAGATGGGTCCTAATTGGGCGATTTGCCACTGATACAGGAGGCTACTCATGAACGAAAGACACTTAACAGGTGCTGCTTCTGAAATGCTAGTAGCCTACAAATTTACCAAAGCTTGGTACACTGTCTATTACCCTATGCTGACTCAGAGTAAAGAGGACTTGGTTGTATGTAAGGATGGTAAATTTAGTAAGGTTCAGGt                                                                                                                                               <  1:397556/272‑1 (MQ=255)`  
                                                                                                                                                             `gTACCGAAGAGATTGCTCAGGTGGTCATTGAGACCGCACAAGAAGCGATGCGCTGGGTTGGAGACCACTGGAACTTCCGGTGTCTTCTGGATACCGAAGGTAAGATTGGTCCTAATTGGGTGATTTGCCACTGATACAGGAGGCTACTCATGAACGAAAGACACTTAACAGGTGCTGCTTCTGAAATGCTAGTAGCCTACAAATTTACCAAAGCTGGGTACACTGTCTATTACCCTATGCTGACTCAGAGTAAAGAGGACTTGGTTGTATGTAAGGATGGTAAATTTAGTAAGGTTCAGGt                                                                                                                                               <  1:661348/301‑1 (MQ=255)`  
                                                                                                                                                             `gTACCGAAGAGATTGCTCAGGTGGTCATTGAGACCGCACAAGAAGCGATGCGCTGGGTTGGAGACCACTGGAACTTCCGGTGTCTTCTGGATACCGAAGGTAAGATGGGTCCTAATTGGGCGATTTGCCACTGATACAGGAGGCTACTCATGAACGAAAGACACTTAACAGGTGCTGCTTCTGAAATGCTAGTAGCCTACAAATTTACCAAAGCTGGGTACACTGTCTATTACCCTATGCTGACTCAGAGTAAAGAGGACTTGGTTGTCTGTAAGGATGGTAAATTTAGTAAGGTTCAGGt                                                                                                                                               >  1:451094/1‑301 (MQ=255)`  
                                                                                                                                                             `gTACCGAAGAGATTGCTCAGGTGGTCATTGAGACCGCACAAGAAGCGATGCGCTGGGTTGGAGACCACTGGAACTTCCGGTGTCTTCTGGATACCGAAGGTAAGATGGGTCCTAATTGGGCGATTTGCAACTGATACAGGAGGCTACTCATGAACGAAAGACACTTAACAGGTGCTGCTTCTGAAATGCTAGTAGCCTACAAATTTACCAAAGCTGGGTACACTGTCTATTACCCTATGCTGACTCAGAGTAAAGAGGACTTGGTTGTATGTAAGGATGGTAAATTTAGTAAGGTTCAGGt                                                                                                                                               >  1:733266/1‑301 (MQ=255)`  
                                                                                                                                                             `gTACCGAAGAGATTGCTCAGGTGGTCATTGAGACCGCACAAGAAGCGATGCGCTGGGTTGGAGACCACTGGAACTTCCGGTGTCTTCTGGATACCGAAGGTAAGATGGGTACTAATTGGGCGATTTGACACTGATACAGGAGGCTACTCATGAACGAAAGACACTTAACAGGTGCTGCTTCTGAAATGCTAGTAGCCTACAAATTTACCAAAGCTGGGTACACTGTCTATTACCCTATGCTGACTCAGAGTAAAGAGGACTTGGTTGTATGTAAGGATGGTAAATTTAGTaactgtctctt                                                                                                                                               >  1:654924/1‑292 (MQ=255)`  
                                                                                                                                                                   `aaGAGATTGCTCAGGTGGTCATTGGGACCGCACAAGAAGCGATGCGCTGGGTTGGAGACCACTGGAACTTCCGGTGTCTTCTGGATACCGAAGGTAAGATGGGTCCTAATTGGGCGATTTGCCACTGATACAGGAGGCTACTCATGAACGAAAGACACTTAACAGGTGCTGCTTCTGAAATGCTAGTAGCCTACAAATTTACCAAAGCTGGGTACACTGTCTATTACCCTATGCTGACTCAGAGTAAAGAGGACTTGGTTGTATGTAAGGATGGTAAATTTAGTAAGGTTCAGGTTAAAAc                                                                                                                                         <  1:130831/301‑1 (MQ=255)`  
                                                                                                                                                                           `gCTCAGGTGGTCATTGAGACCGCACAAGAAGCGATGCGATGGGTTGGAGACCACTGGAACTTCCGGTGTCTTCTGGATACCGAAGGTAAGATGGGTCCTAATTGGGCGATTGGCCACTGATACAGGAGGCTACTCATGAACGAAAGACACTTAACAGGTGCTGCTTCTGAAATGCGAGTAGACTACAAATTTACCAAAGCTGGGTACACTGTCTATTACCATATGCTGACACAGAGTAAAGAGGACTTGGTGGTATGTAAGGAGGGTAAATTTAGTAAGGTTCAGGTTAAAACAGCCACaa                                                                                                                                 >  1:668790/1‑301 (MQ=255)`  
                                                                                                                                                                                `ggtggtCATTGAGACCGCACAAGAAGCGATGCGCTGGGTTGGAGACCACTGGAACTTCCGGTGTCTTCTGGATACCGAAGGTAAGATGGGTCCTAATTGGGCGATTTGCCACTGATACAGGAGGCTACTAATGAACGAAAGACACTTAACAGGTGCTGCTTCTGAAATGCTAGTAGCCTTCAAATTTACCAAAGCTGGGTACACTGTCTATTACCCTATGCTGACTCAGAGTAAAGAGGACTTGGTTGTATGTAAGGATGGTAAATTTAGTAAGGTTCAGGTTAAAACAGCCACAACGGtt                                                                                                                            >  1:243416/1‑301 (MQ=255)`  
                                                                                                                                                                                    `gtCATTGAGACCGCACAAGAAGCGATGCGCTGGGTTGGAGACCACTGGAACTTCCGGTGTCTTCTGGATACCGAAGGTAAGATGGGTCCTAATTGGGCGATTTGCCACTGATACAGGAGGCTACTCATGAACGAAAGACACTTAACAGGTGCTGCTTCTGAAATGCTAGTAGCCTACAAATTTACCAAAGCTGGGTACACTGTCTATTACCCTATGCTGACTCAGAGTAAAGAGGACTTGGTTGTATGTAAGGATGGTAAATTTAGTAAGGTTCAGGttctgtctcttatacacatctccg                                                                                                                        >  1:581877/1‑279 (MQ=255)`  
                                                                                                                                                                                      `aaTTGAGACCGCACAAGAAGCGATGCGCTGGGTTGGAGACCACTGGAACTTCCGGTGTCTTCTGGATACCGAAGGTAAGATGGGTCCTAATTGGGCGATTTGCCACTGATACAGGAGGCTACTCATGAACGAAAGACACTTAACAGGTGCTGCTTCTGAAATGCTAGTAGCCTACAAATTTACCAAAGCTGGGTACACTGTCTATTACCCTATGCTGACTCAGAGTAAAGAGGACTTGGTTGTATGTAAGGATGGTAAATTTAGTAAGGTTCAGGTTAAAACAGCCACAACGGTTCAAAcc                                                                                                                      <  1:253189/300‑1 (MQ=255)`  
                                                                                                                                                                                           `agaCCGCACAAGAAGCGATGCGCTTGGTTGGAGACCACTGGCACTTCCGGTGTCTTCTGGATACCGAAGGTAAGATGGGTCCTAATTGTGCGATTTGCCACTGATACAGGAGGCTACTCATGAACGAAAGACACTTAACAGGTGCTGCTTCTGAAATGCTAGTAGCCTACAAATTTACCAAAGCTGGGTACACTGTCTATTACCCTATGCTGACTCAGAGTAAAGAGGACTTGGTTGTATGTAAGGATGGTAAATTTATTAAGGTTCAGGTTAAAACAGCCACAACGGTTCAAACCAacac                                                                                                                 >  1:464809/1‑301 (MQ=255)`  
                                                                                                                                                                                             `aCCGCACAAGAAGCGATGCGCTGGGTTGGAGACCACTGGAACTTCCGGTGTCTTCTGGATACCGAAGGTAAGATGGGTCCTAATTGGGCGATTTGCCACTGATACAGGAGGCTACTCATGAACGAAAGACACTTAACAGGTGCTGCTTCTGAAATGCTAGTAGCCTACAAATTTACAAAAGCTGGGTACACTGTCTATTACCCTATGCTGACTAAGAGTAAAGAGGACTTGGTTGTATGTAAGGATGGTAAATTTAGTAAGGTTCAGGTTAAAACAGCCACAACGGTTcctgtctcttata                                                                                                               >  1:799388/1‑289 (MQ=255)`  
                                                                                                                                                                                              `tgtgtataagagacaggcgcTGGGTTGGAGACCACTGGAACTTCCGGTGTCTTCTGGATACCGAAGGTAAGATGGGTCCTAATTGGGCGATTTGCCACTGATACAGGAGGCTACTCATGAACGAAAGACACTTAACAGGTGCTGCTTCTGAAATGCTAGTAGCCTACAAATTTACCAAAGCTGGGTACACTGTCTATTACCCTATGCTGACTCAGAGTAAAGAGGACTTGGTTGTATGTAAGGATGGTAAATTTAGTAAGGTTCAGGTTAAAACAGCCACAACGGTTCAAACCAACACAgg                                                                                                              <  1:640534/285‑1 (MQ=255)`  
                                                                                                                                                                                              `ctgCACAAGAAGCGAGGCGCTGGGTTGGAGACCACTGGAACTTCCGGTGTCTTCTGGATACCGAAGGTAAGATGGGTCCTAATTGGGCGATTTGCCACTGATACAGGAGGCTACTCATGAACGAAAGACACTTAACAGGTGCTGCTTCTGAAATGCTAGTAGCCTATAAATTTACCAAAGCTGGGTACACTGTCTATTACCCTATGCTGACTCAGAGTAAAGAGGACTTGGTTGTATGTAAGGATGGTAAATTTAGTAAGGTTCAGGTTAAAACAGCCACAACGGTTCAAACCAACACAgg                                                                                                              <  1:231761/299‑1 (MQ=255)`  
                                                                                                                                                                                                `gtgggctcggagatgtgtataagagacagccACTGGAACTTCCGGTGTCTTCTGGATACCGAAGGTAAGATGGGTCCTAATTGGGCGATTTGCCACTGATACAGGAGGCTACTCATGAACGAAAGACACTTAACAGGTGCTGCTTCTGAAATGCTAGTAGCCTACAAATTTACCAAAGCTGGGTACACTGTCTATTACCCTATGCTGACTCAGAGTAAAGAGGACTTGGTTGTATGTAAGGATGGTAAATTTAGTAAGGTTCAGGTTAAAACAGCCACAACGGTTCAAACAAACACAGgag                                                                                                            <  1:267883/272‑1 (MQ=255)`  
                                                                                                                                                                                                     `agaagCGATGCGCTGGGTTGGAGACCACTGTAACTTCCGGTGTCTTCTGGATACCGAAGGTAAGATGGGTCCTAATTGGGCGATTTGCCACTGATACAGGAGGCTACTCATGAACTAAAGACACTTAACAGGTGCTGCTTCTGAAATGCTAGTAGCCTACAAATTTACCAAAGCTGGGTACACTGTCTATTACCCTATGCTTACTCAGAGTAAAGAGGACTTGGTTGTATGTAAGGATGGTAAATTTAGTAAGGTTCAGGTTAAAACAGCCACAACGGTTCAAACCAACACAGGAGATGcc                                                                                                       <  1:157918/301‑1 (MQ=255)`  
                                                                                                                                                                                                                   `taagagacagccACTGGAACTTCCGGTGTCTTCTGGATACCGAAGGTAAGATGGGTCCTAATTGGGCGATTTGCCACTGATACAGGAGGATACTAATGAACGAAAGACACTTAACAGGTGCTGCTTCTGAAATGCTAGTAGCCTACAAATTTACCAAAGCTGGGTACACTGTCTATTACCCTATGCTGACTCAGAGTAAAGAGGACTTGGTTGTATGTAAGGATGGTAAATTTAGTAAGGTTCAGGTTAAAACAGCCACAACGGTTCAAACCAACACAGGAGATGCCAAGCAGGTTAGGCt                                                                                         <  1:141172/291‑1 (MQ=255)`  
                                                                                                                                                                                                                   `gggTTGGAGACCACTGGAACTTCCGGTGTCTTCTGGATACCGAAGGTAAGATGGGTCCTAATTGGGCGATTTGCCACTGATACAGGAGGCTACTCATGAACGAAAGACACTTAACAGGTGCTGCTTCTGAAATGCTAGTAGCCTACAAATTTACCAAAGCTGGGTACACTGTCTATTACCCTATGCTGACTCAGAGTAAAGAGGACTTGGTTGTATGTAAGGATGGTAAATTTAGTAAGGTTCAGGTTAAAACAGCCACAACGGTTCAAACCAACACAGGAGATGCCAAGCAGGTTAGGCt                                                                                         >  1:785840/1‑301 (MQ=255)`  
                                                                                                                                                                                                                   `gggTTGGAGACCACTGGAACTTCCGGTGTCTTCTGGATACCGAAGGTAAGATGGGTCCTAATTGGGCGATTTGCCACTGATACAGGAGGCTACTCATGAACGAAAGACACTTAACAGGTGCTGCTTCTGAAATGCTAGTAGCCTACAAATTTACCAAAGCTGGGTACACTGTCTATTACCCTATGCTGACTCAGAGTAAAGAGGACTTGGTTGTATGTAAGGATGGTAAATTTAGTAAGGTTCAGGTTAAAACAGCCACAACGGTTCAAACCAACACAGGAGATGCCAAGCAGGTTAGGCt                                                                                         >  1:589149/1‑301 (MQ=255)`  
                                                                                                                                                                                                                   `gggTTGGAGACCACTGGAACGTCCGGTGTCTTCTGGATACCGAAGGTAAGATGGGTCCTAATTGGGCGATTTGCCACTGATACAGGAGGCTACTCATGAACGAAAGACACTTAACAGGTGCTGCTTCTGAAATGCTAGTAGCCTACAAATTTACCAAAGCTGGGTACACTGTCTATTACCCTATGCTTACTCAGAGTAAAGAGGACTTGGTTGTATGTAAGGATGGTAAATTTAGTAAGGTTCAGGTTAAAACAGCCACAACGGTTCAAACCAACACAGGAGATTCCAAGCAGGTTAGGCt                                                                                         <  1:326434/301‑1 (MQ=255)`  
                                                                                                                                                                                                                     `gTTGGAGACCACTGGAACTTCCGGTGTCTTCTGGATACCGAAGGTAAGATGGGTCCTAATTGGGCGATTTGCCACTGATACAGGAGGCTACTCATGAACGAAAGACACTTAACAGGTGCTGCTTCTGAAATGCTAGTAGCCTACAAATTTACCAAAGCTGGGTACACTGTCTATTACCCTATGCTGACTCAGAGTAAAGAGGACTTGGTTGTATGTAAGGATGGTAAATTTAGTAAGGTTCAGGTTAAAACAGCCACAACGGTTCAAACCAACACAGGAGATGccctgtatcttatacaca                                                                                       >  1:384425/1‑285 (MQ=255)`  
                                                                                                                                                                                                                        `ggAGAACACTGGAACTTCCGGTGTCTTCTGGATACCGAAGGTAAGATGGGTCCTAATTGGGTGATTTGCCACTGATACATGAGGCTACTCATGAACGAAAGACACTTAACAGGTGCTGCTTCTGAAATGCTAGTAGCCTACAAATTTACCAAAGCTGGGTACACTGTCTATTACCCTATACTGACTCAGAGTAAAGAGGACTTGGTTGTATGTAAGGATGGTAAATTTAGTAAGGTTCAGGTTAAAACAGCCACAACGGTTCAAACCAACACAGGAGATGCCAAGCAGGTTAGGCTAGGTg                                                                                    <  1:808359/301‑1 (MQ=255)`  
                                                                                                                                                                                                                                     `ggctcggagatgtgtataagggacagggTAAGATGGGTCCTAATTGGGCGATTTGCCACTGATACAGGAGGCTACTCATGAACGAAAGACACTTAACAGGTGCTGCTTCTGAAATGCTAGTAGCCTACAAATTTACCAAAGCTGGGTACACTGTCTATTACCCTATGCTGACTCAGAGTAAAGAGGACTTGGTTGTATGTAATGATGGTAAATTTAGTAAGGTTCAGGTTAAAACAGCCACAACGGTTCAAACCAACACAGGAGATGCCAAGCAGGTTAGGCTAGGTTGATGCGGTAGGTc                                                                       <  1:793729/275‑1 (MQ=255)`  
                                                                                                                                                                                                                                     `aagagacaggtCTTCTGGATACCGAAGGTAAGATGGGTCCTAATTGGGCGATTTTCCACTGATACAGGAGGCTACTCATGAACGAAAGACACTTAACAGGTGCTGCTTCTGAAATGCTAGTAGCCTACAAATTTACCAAAGCTGGGTACACTGTCTATTACCCTATGCTGACTCAGAGTAAAGAGGACTTGGTTGTATGTAAGGATGGTAAATTTAGTAAGGTTCAGGTTAAAACAGCCACAACGGTTCAAACCAACACAGGAGATGCCAAGCAGGTTAGGCTAGGTGGATGCGGTAGGTc                                                                       <  1:718878/292‑1 (MQ=255)`  
                                                                                                                                                                                                                                     `aCTTCCGGTATCTTCTGGATACCGAAGGTAAGATGGGTCCTAATTGGGCGATTTGCCACTGATACAGGAGGCTACTCATGAACGAAAGACACTTAACAGGTGCTGCTTATGAAATGCTAGCAGCCTACAAATTTACCAAAGCTGGGTACACTGTCTATTACCCTATGCTGACTCAGAGTAAAGAGGACTTGGTTGTATGTAAGGATGGTAAATTTAGTAAGGTTAAGGTTAAAACAGCCACAACGGTTCAAACCAACACAGGAGATGCCAAGCAGGTTAGGCTAGGTGGATGCGGTAGGTc                                                                       <  1:692057/301‑1 (MQ=255)`  
                                                                                                                                                                                                                                      `cTTCCGGTGTCTTCTGGATACCGAAGGTAAGATGGGTCCTAATTGGGCGATTTGCCACTGATACAGGAGGCTACTCATGAACGAAATACACTTAACAGGTGCTGCTTCTGAAATGCTAGTAGCCTACAAATTTACCAAAGCTGGGTACACTGTCTATTACCCTATGCTGACTCAGAGTAAAGATGACTTGGTTGTATGTAAGGATGGTAAATTTAGTAAGGTTCAGGTTAAAACAGCCACAACGGTTCAAACCAACACAGGAGATGCCAAGCAGGTTAGGCTAGGTGGATGCGGTAGGTcc                                                                      >  1:776432/1‑301 (MQ=255)`  
                                                                                                                                                                                                                                          `cGGTGTCTTCTGGATACCGAATGTAATATTGGTCCTAATTTGGCGATTTGCCACTGATACAGGAGGCTACTCATGAACGAAAGACACTTAACAGGTGCTGCTTCTGAAATGCTAGTAGCCTACAAATTTACCAAAGCTGGGTACACTGTCTATTACCCTATGCTGACTCAGAGTAAAGAGGACTTGGTTGTATGTAAGGATGGTAAATTTAGTAAGGTTCAGGTTAAAACAGCCACAACGGTTCAAACCAACACAGGAGATGCCAAGCAGGTTAGGCTAGGTGGATGCGGTAGGTCCGAat                                                                  <  1:683947/301‑1 (MQ=255)`  
                                                                                                                                                                                                                                            `gtgtCTTCTGGATACCGAAGGTAAGATGGGTCCTAATTGGGCGATTTGCCACTGATACAGTAGGCTACTCATGAACGAAAGACACTTAACAGGTGCTGCTTCTGAAATGCTAGTAGCCTACAAATTTACCAAAGCTGGGTACACTGTCTATTACCCTATGCTGACTCAGAGTAAAGAGGACTTGGTTGTATGTAAGGATGGTAAATTTAGTAAGGTTCAGGTTAAAACAGCCACAACGGTTCAAACCAACACAGGAGATGCCAAGCAGGTTAGGCTAGGTGGATGCGGTAGGTCCGAatat                                                                <  1:300286/301‑1 (MQ=255)`  
                                                                                                                                                                                                                                                `ctcggagatgtgtataagagacagggTCCTAATTGGGCGATTTGCCACTGATACAGGAGGCTACTCATGAACGAAAGACACTTAACAGGTGCTGCTTCTGAAATGCTAGTAGCCTACAAATTTACCAAAGCTGGGTACACTGTCTATTACCCTATGCTGACTCAGAGTAAAGAGGACTTGGTTGTATGTAAGGATGGTAAATTTAGTAAGGTTCAGGTTAAAACAGCCACAACGGTTCAAACCAACACAGGAGATGCCAAGCAGGTTAGGCTAGGTTGATGCGGTAGGTCCGAATATAAgg                                                            <  1:147847/278‑1 (MQ=255)`  
                                                                                                                                                                                                                                                 `gatgtgtataagagacagaGATGGGTCCTAATTGGGCGATTTGCCACTGATACAGGAGGCTACTCATGAACGAAAGACACTTAACAGGTGCTGCTTCTGAAATGCTAGTAGCCTACAAATTTACCAAAGCTGGGTACACTGTCTATTACCCTATGCTGACTCAGAGTAAAGAGGACTTGGTTGTATGTAAGGATGGTAAATTTAGTAAGGTTCAGGTTAAAACAGCCACAACGGTTCAAACCAACACAGGAGATGCCAAGCAGGTTAGGCTAGGTGGATGCGGTAGGTCCGAATATAAGGa                                                           <  1:498768/283‑1 (MQ=255)`  
                                                                                                                                                                                                                                                       `aTACCGAAGGTAAGATGGGTCCTAATTGGGCGATTTGCCACTGATACAGGAGGCTACTCATGAACGAAAGACACTTAACAGGTGCTGCTTCTGAAATGCTAGTAGCCTACAAATTTACCAAAGCTGGGTACACTGTCTATTACCCTATGCTGACTCAGAGTAAAGAGGACTTGGTTGTATGTAAGGATGGTAAATTTAGTAAGGTTCAGGTTAAAACAGCAACAACGGTTCAAACCAACACAGGAGATGCCAAGCAGGTTAGGCTAGGTGGATGCGGTAGGTCCGAATATAAGGCTgtctc                                                     >  1:562194/1‑297 (MQ=255)`  
                                                                                                                                                                                                                                                                                                  `aTACAGGAGGCTACTCATGAACGAAAGACACTTAACAGGTGCTGCTTCTGAAATGCTAGTAGCCTACAAATTTACCAAAGCTGGGTACACTGTCTATTACCCTATGCTGACTCAGAGTAAAGAGGACTTGGTTGTATGTAAGGATGGTAAATTTAGTAAGGTTCAGGTTAAAACAGCCACAACGGTTCAAACCAACACAGGAGATGCCAAGCAGGTTAGGCTAGGTGGATGCGGTAGGTCCGAATATAAGGAAGGAGACTTTGACATTCTTGCGGTTGTGGTTGACGAAGATGTGCTTAtt          >  1:556730/1‑301 (MQ=255)`  
                                                                                                                                                                                                                                                                                                   `tACAGGAGGCTACTCATGAACGAAAGACACTTAACAGGTGCTGCTTCTGAAATGCTAGTAGCCTACAAATTTACCAAAGCTGGGTACACTGTCTATTACCCTATGCTGACTCAGAGTAAAGAGGACTTGGTTGTATGTAAGGATGGTAAATTTAGTAAGGTTCAGGTTAAAACAGCCACAACGGTTCAAACCAACACAGGAGATGCCAAGCAGGTTAGGCTAGGTGGATGCGGTAGGTCCGAATATAAGGATGGAGACTTTGACATTCTTGCGGTTGTGGTTGACGAAGATGTGCTTAttt         >  1:570471/1‑301 (MQ=255)`  
                                                                                                                                                                                                                                                                                                    `aCAGGAGGCTACTCATGAACGAAAGACACTTAACAGGTGCTGCTTCTGAAATGCTAGTAGCCTACAAATTTACCAAAGCTGGGTACACTGTCTATTACCCTATGCTGACTCAGAGTAAAGAGGACTTGGTTGTATGTAAGGATGGTAAATTTAGTAAGGTTCAGGTTAAAACAGCCACAACGGTTCAAACCAACACAGGAGATGCCAAGCAGGTTAGGCTAGGTGGATGCGGTAGGTCCGAATATAAGGATGGAGACTTTGACATTCTTGCGGTTGTGGTTGACGAAGATGTGCTTAtttt        >  1:407055/1‑301 (MQ=255)`  
                                                                                                                                                                                                                                                                                                     `cgggaggCTACTCATGAACGAAAGACACTTAACAGGTGCTGCTTCTGAAATGCTAGTAGCCTACAAATTTACCAAAGCTGGGTACACTGTCTATTACCCTATGCTGACTCAGAGTAAAGAGGACTTGGTTGTATGTAAGGATGGTAAATTTAGTAAGGTTCAGGTTAAAACAGCCACAACGGTTCAAACCAACACAGGAGATGCCAAGCAGGTTAGGCTAGGTGGATGCGGTAGGTCCGAATATAAGGATGGAGACTTTGACATTCTTGCGGTTGTGGTTGACGAAGATGTGCTTAttttt       >  1:214884/3‑300 (MQ=255)`  

                                                                                                                                                                                                                                                                                                           `|`                                                                                                                                                                                                                                                                                                             
`GCCTTGAATACCCTACTGCAATCTGCTGGTGCTCTCATCTGCAAACTGTGGATTATCAAGACCGAAGAGATGCTCGTAGAGAAAGGCTTGAAGCATGGCTGGGATGGGGACTTTGCGTACATGGCATGGGTACATGATGAAATCCAAGTAGGCTGCCGTACCGAAGAGATTGCTCAGGTGGTCATTGAGACCGCACAAGAAGCGATGCGCTGGGTTGGAGACCACTGGAACTTCCGGTGTCTTCTGGATACCGAAGGTAAGATGGGTCCTAATTGGGCGATTTGCCACTGATACAGGAGGCTACTCATGAACGAAAGACACTTAACAGGTGCTGCTTCTGAAATGCTAGTAGCCTACAAATTTACCAAAGCTGGGTACACTGTCTATTACCCTATGCTGACTCAGAGTAAAGAGGACTTGGTTGTATGTAAGGATGGTAAATTTAGTAAGGTTCAGGTTAAAACAGCCACAACGGTTCAAACCAACACAGGAGATGCCAAGCAGGTTAGGCTAGGTGGATGCGGTAGGTCCGAATATAAGGATGGAGACTTTGACATTCTTGCGGTTGTGGTTGACGAAGATGTGCTTATTTTCACATG  >  NC_001604.1/16177‑16775`

  

Alignment Legend

Aligned base mismatch/match (shaded by quality score): `ATCG/ATCG` `< 3 ≤` `ATCG/ATCG` `< 18 ≤` `ATCG/ATCG` `< 29 ≤` `ATCG/ATCG` `< 37 ≤` `ATCG/ATCG` `< 38 ≤` `ATCG/ATCG`

Unaligned base: `atcg`    Masked matching base: `atcg`    Alignment gap: `‑`    Deleted base: `‑`

Reads not counted as support for junction

read\_name Not counted due to insufficient overlap past the breakpoint.

read\_name Not counted due to not crossing MOB target site duplication.
